# Differences in disciplinary concentration and linguistic complexity between scientific posts on Bluesky and X

**DOI:** 10.3389/frma.2026.1912970

**Published:** 2026-09-28

**Authors:** Piotr Religa, Michel-Edwar Mickael, Marzena Łazarczyk, Norwin Kubick, Jarosław Olav Horbańczuk, Biniyam Tsegaye, Mariusz Sacharczuk, Atanas G. Atanasov

**Affiliations:** 1 Department of Medicine, Karolinska Institute, Solna, Sweden; 2 Institute of Genetics and Animal Biotechnology of the Polish Academy of Sciences, Magdalenka, Poland; 3 Department of Biology, Institute of Plant Science and Microbiology, University of Hamburg, Hamburg, Germany; 4 Patient Safety and Digital Health Group, Danube Private University, Fakultät Medizin/Zahnmedizin, Krems-Stein, Austria; 5 Department of Biochemistry, Saveetha Medical College and Hospital, Saveetha Institute of Medical and Technical Sciences, Chennai, Tamil Nadu, India; 6 Ludwig Boltzmann Institute Digital Health and Patient Safety, Medical University of Vienna, Vienna, Austria

**Keywords:** Bluesky, data analysis, health information, social media, twitter

## Abstract

Several social media platforms provide a route for communication about scientific topics. X, formerly known as Twitter, has been shown to offer researchers an opportunity to exchange scientific information with audiences extending well beyond the research community. In recent years another platform, Bluesky, has emerged and appears to encourage scientific discourse, and a wave of migration of researchers from X to Bluesky has been documented in survey data, poll responses and altmetric records. Whether scientific claims are described differently on the two platforms has not been fully studied. We collected 8,697 Bluesky posts and 3,48,900 X posts mentioning the term science over a matched observation window, restricted in both cases to English-language text written by the posting account. A combination of data analysis and natural language processing tools was then used to compare the discourse on the two platforms, quantifying disciplinary composition, field concentration, cross-disciplinary reference and linguistic register, with effect sizes reported alongside every test and correction applied for multiple comparisons. The two platforms carried posts across a similar range of science-related topics, including health and medicine, biology and life sciences, and engineering and technology. However, posts on X were easier to read (Flesch–Kincaid grade 9.07 against 10.02), addressed the reader more directly (second-person pronouns 0.0153 against 0.0093) and were less complex (complex word density 0.092 against 0.108). Posts on Bluesky were longer (30.6 against 28.7 words; 15.98 against 14.54 words per sentence), more concentrated within single disciplines (mean field isolation 0.6882 against 0.6200), higher in first-person pronoun use (0.0255 against 0.0208) and more often classified as academic (16.6% against 12.7%). These patterns are consistent with greater peer-oriented scientific discourse on Bluesky and greater audience-oriented dissemination on X, although audience composition was not measured directly.

## 1 Introduction

The landscape of scientific communication has undergone a fundamental transformation over the past two decades, as social media platforms have dismantled traditional barriers between researchers and diverse public audiences (Phogat et al., 2025). Social media enables scientists to communicate directly with wider audiences in real time, facilitating two-way engagement that moves beyond the unidirectional knowledge transfer model of earlier public science initiatives, while expanding possibilities for citizens worldwide to share knowledge and interact about scientific advancements (Scheufele, 2013). Recent pandemics accelerated this transformation, underscoring the importance of accurate science communication via social media channels to counter misinformation and demonstrating that scientists could mobilize rapidly to share emerging findings and guide public understanding (Pollett and Rivers, 2020). Yet this democratization of access cuts both ways: while social media creates opportunities for scientists to engage directly with publics and bypass traditional gatekeepers, it also exposes scientific discourse to commercial pressures, algorithmic distortion, and the polarizing dynamics that characterize broader social media ecosystems (Recuero, 2025).

X, formerly known as Twitter, emerged as a particularly influential platform for scientific discourse, establishing itself as what many researchers consider essential infrastructure for modern academic communication (Stewart, 2015). The platform became a prominent social networking site for science communication, with roughly half its U. S. users consuming news on the platform, creating a venue where scientists could reach both specialist and general audiences simultaneously (Zhang M. et al., 2025). X has accelerated scientific communication and dissemination of research findings, evaluation and discussion of scientific work, and collaboration with other scientists. On the academic level, researchers use X primarily for advertising publications and research updates, similar to marketing and public relations efforts, though the platform also facilitates conference participation, peer networking, and public engagement (Chan et al., 2022). Studies of academic ecologists and evolutionary biologists found that researchers with more than 1,000 followers reached audiences composed primarily of policymakers, journalists, and other nonscientists rather than only colleagues (Cote and Darling, 2018).

Despite their decentralized and open architecture, social media platforms often do not present fully equitable environments, with research revealing systematic biases favoring well-resourced institutions, Anglophone countries, and sensational topics over incremental scholarship (Portman et al., 2025). Algorithmic curation, character constraints, and engagement metrics create pressures that influence science visibility, potentially privileging controversy, novelty, and emotional resonance over methodological rigor or nuanced complexity. X underwent profound changes that prompted various researchers to seek alternative venues, with Bluesky emerging as the primary destination for scientific migration (Biever, 2025; Shiffman and Wester, 2025). Comparisons of the two platforms have addressed user counts, posting volume, engagement and altmetric mentions, and survey-based perceptions of platform suitability (Arroyo-Machado et al., 2025; Stone and Schwartz, 2025). Adoption has been reported at the level of specialist communities rather than of individual accounts (Gussow, 2025), assisted by starter packs that supply a new account with the follower base of an established community (Balduf et al., 2025). An earlier attempt to relocate academic discourse to Mastodon did not sustain engagement despite strong connectivity among the migrants (Wang et al., 2026), and migration across platforms has been described as a staged process (Jeong et al., 2024). The internal structure of the discourse itself has not been fully studied, including the distribution of posts across disciplines, the rate at which single posts bridge disciplinary boundaries, and the linguistic register in which scientific claims are expressed.

To address these questions, we conducted a comparative analysis of scientific discourse on Bluesky and X, examining posts mentioning the keyword science using disciplinary classification, co-occurrence networks, field concentration indices, readability measures, and a composite index of scholarly linguistic markers. Both corpora were restricted to English-language text written by the posting account, so that the two platforms are compared on material of the same kind. This combination of measures addresses the disciplinary composition of each platform alongside the register in which its posts are written, covering terminology, cross-disciplinary reference and linguistic complexity.

## 2 Methods

### 2.1 Data import and cleaning

Data from X was collected using the Fedica Analytics Platform (https://fedica.com/), which facilitated the extraction of posts, likes, mentions, and other engagement metrics, and posts published within the observation window that included the term science were retrieved. Bluesky data were collected through a custom retrieval pipeline in a Python environment using the atproto SDK, pandas for data structuring and the re library for text sanitization, with a research client authenticated using app-specific credentials. Retrieval proceeded through session establishment with the Bluesky XRPC endpoint, keyword-based queries targeting the term science, and recursive cursor-based pagination to bypass the 100-post API limit, covering the same observation window as the X posts. For each qualifying post a multi-dimensional record was constructed capturing user metadata, temporal metadata, full text content and engagement metrics, and textual data underwent sanitization using a regular expression to remove non-printable control characters and null bytes. Both retrievals used the same query term and the same observation window. The X corpus was restricted to authored posts and replies so that both corpora comprise text written by the posting account rather than forwarded content; no equivalent restriction was required on Bluesky, as the search endpoint returns authored records only and reposts are stored as separate records that were not retrieved, and both corpora were restricted to English-language posts, as the readability indices implemented in textstat and the spaCy model used for part-of-speech tagging are calibrated for English. The observation window was selected to capture a continuous period of routine academic activity, falling between the end of the northern-hemisphere teaching semester and the summer conference season, and no globally reported scientific announcement is known to have occurred within it, so that the retrieved discourse is unlikely to be organized around a single focal event. Posts were imported using pandas v2.1.1, which implements the data structures described by McKinney (McKinney, 2010). Only the primary textual content fields were retained, and posts with missing or empty text were removed. URLs, mentions and hashtags were removed from the cleaned text used for readability scoring and part-of-speech tagging, using regular expressions, and whitespace was normalized; the number of hashtags per post was counted on the original text before cleaning and is reported among the register measures, while punctuation relevant for sentence segmentation was retained. No stemming or lemmatisation was applied, as these procedures can distort discipline-specific terminology and bias readability metrics (Siino et al., 2024).

### 2.2 Rule-based scientific field assignment

Scientific domains were assigned using a dictionary-based, multi-label classification strategy. Established disciplinary taxonomies assign a field to a publication using venue metadata, reference lists or full text, none of which is available for a post of a few dozen words, and they assign a single field where a post may refer to more than one. Keyword dictionaries were therefore curated manually for ten scientific fields, drawing on established disciplinary vocabularies and prior science-communication literature. Keywords included both unigrams and multi-word expressions. Each post was scanned for keyword matches at word boundaries, so that a keyword is counted only where it occurs as a complete word rather than as a substring of a longer word, and the complete dictionaries are provided in the Supplementary Material. To validate this approach, a validation sample of 99 posts was drawn at random from the pooled corpus and coded independently by two annotators, who assigned each post to one or more of the ten disciplinary categories, or to none, while blind to the platform of origin and to the dictionary output. Coding followed a written manual that defines each category and specifies that a discipline is marked only where the post discusses a subject belonging to that field, and not where a scientific term is used as a metaphor, an insult, or in reference to scientists, funding or institutions. Agreement between annotators was quantified using Cohen's kappa, and the consensus labels were compared with the automated assignment. Posts were allowed to match multiple fields, so that a post referring to two disciplines is recorded as belonging to both rather than being assigned to one of them. Field frequencies were computed by summing all field assignments on a platform and expressing each field as a proportion of that total, so that the reported shares are proportions of field assignments rather than of posts. Word clouds showing the most frequent nouns per discipline were generated using the WordCloud library with a maximum of 50 words displayed.

### 2.3 Cross-disciplinary co-occurrence networks

For posts assigned to multiple scientific fields, all unordered pairs of co-occurring fields were generated and aggregated into weighted adjacency matrices, and networks were constructed using NetworkX, with nodes representing scientific domains and edge weights representing normalized co-occurrence frequency (Bouwer et al., 2021). Edges were retained where the co-occurrence rate exceeded a fixed proportion of labeled posts, so that the threshold is comparable across corpora of different size, and the node set was fixed at all ten fields so that network density shares a denominator between platforms. Network density, average clustering coefficient and the number of connected components were computed for each platform. Disciplinary breadth was quantified by the interdisciplinary post rate, defined as the proportion of labeled posts referencing more than one field, and by the Shannon entropy and Simpson index of the field distribution on each platform.

### 2.4 Field concentration

Field isolation was computed per discipline as the proportion of posts within that discipline that reference that discipline alone. Isolation indices were calculated separately for each platform and compared across the ten disciplines, and a composite score was computed as the unweighted arithmetic mean of the ten field-specific isolation proportions, so that each discipline contributes equally regardless of its size. The Composite Disciplinary Concentration Index aggregates four indicators into a single score ranging from 0 to 1: mean isolation (weight 0.3), the interdisciplinary rate subtracted from one (0.3), the network density subtracted from one (0.2), and the average clustering coefficient (0.2). Each indicator already lies between zero and one, so the two inverted terms are linear complements rather than rescaled quantities, and the index is reported both under these weights and under equal weighting of the four indicators. The index describes the disciplinary concentration of post content.

### 2.5 Linguistic complexity and readability

Readability metrics were computed using textstat v0.7.3, including Flesch Reading Ease, Flesch-Kincaid Grade, Gunning Fog Index, SMOG Index, Coleman-Liau Index, Automated Readability Index, Dale-Chall Readability and difficult word counts (Saeedi and Bakhtiar, 2025; Mangilaleng et al., 2025; Rahbe et al., 2025). URLs, mentions and hashtags were removed before any readability index was computed, as these strings are scored as long unfamiliar words by every index. Sentence segmentation was performed using the spaCy sentence boundaries, and posts were classified into accessible, intermediate or academic categories using thresholds combining the Flesch-Kincaid grade, the Gunning Fog Index and the difficult-word count, applied identically to both platforms: a post was classified as academic where the Flesch-Kincaid grade exceeded 14, the Gunning Fog Index exceeded 14 and the difficult-word count exceeded five, and as accessible where the Flesch-Kincaid grade was below 10, the Gunning Fog Index below 10 and the difficult-word count below three, with all remaining posts classified as intermediate. Characters per word, syllables per word, words per sentence, the type-token ratio and the polysyllabic word ratio were computed alongside the readability indices.

### 2.6 Academic terms usage

To operationalise the prevalence of technical language, we used morphological, lexical and structural indicators. Tokens were classified as scientific or technical if they contained domain-typical prefixes or suffixes, using a manually curated list optimized for biomedical and technical discourse, and exact matching was performed against a curated lexicon of academic terms. These features were extracted using spaCy v3.7.2 for part-of-speech tagging, complex words were identified as tokens exceeding eight characters, which is distinct from the difficult-word count reported among the readability measures, where a word is difficult if it does not appear on the Dale-Chall list of familiar words; acronyms were identified as uppercase tokens longer than one character. Case was preserved during tagging, as lowercasing the text before analysis prevents acronym detection and degrades proper-noun tagging. Noun density was computed as the proportion of nouns and proper nouns relative to total tokens, and an aggregate technical score was calculated as the mean of the scientific term, academic word and complex word densities, with noun density reported separately and not entering this score.

### 2.7 Subjectivity and emotional language

We quantified subjectivity and emotional depth using sentiment analysis and grammatical profiling. TextBlob v0.17.1 was used to calculate subjectivity and polarity scores, and we measured the density of first-person and second-person pronouns, emotional verbs, storytelling vocabulary, hedging language and certainty language, extracted by filtering lemmas and part-of-speech tags generated by spaCy. Passive constructions were identified from the dependency parse rather than from a list of auxiliary verbs, and are reported per sentence.

### 2.8 Composite scientificity index

The Composite Scientificity Index aggregates six dimensions, each computed per post and converted to a z-score across the pooled corpus: vocabulary density, methodological language, citation signals, epistemic hedging, argument structure, and emotion suppression. The index is the unweighted mean of the six z-scores, computed with scikit-learn (Kramer, 2016). Equal weighting was adopted in the absence of an established weighting scheme. The index describes the density of scholarly linguistic markers in a post and does not measure the epistemic quality of the underlying claims. Stability was assessed by recomputing the index with each dimension removed in turn, and by an alternative index weighted by the loadings of the first principal component, and the six dimensions are also reported individually.

### 2.9 Sensitivity analysis

To address the difference in size between the two corpora, a sensitivity analysis was conducted using repeated random subsampling. In each of 100 independent iterations, a random sample of X posts equal in size to the Bluesky corpus was drawn without replacement, and eleven key metrics were recomputed on the subsample: field isolation index, interdisciplinary post rate, Shannon H, Simpson diversity, scientific term density, academic word density, complex word density, acronym density, hedging ratio, aggregate technical score and Flesch-Kincaid grade. For each metric the Bluesky point estimate, computed over the full Bluesky corpus rather than over the matched samples used for the post-level comparisons, was compared against the distribution of X subsample values, and the 2.5th and 97.5th percentiles of that distribution are reported as the empirical interval.

### 2.10 Statistical analysis

Post-level comparisons were computed on random samples of 5,000 posts drawn from each platform, so that the two corpora contribute equally, and posts shorter than five tokens were excluded from feature extraction. For each comparison the unit of analysis was identified and the distribution assessed using the Shapiro-Wilk test. Field isolation is calculated once per discipline on each platform, giving ten paired values; with ten pairs a normality test has limited power, and both a paired *t*-test and the Wilcoxon signed-rank test are reported. Readability, linguistic and scientificity measures are calculated per post, and as these distributions departed from normality the Mann-Whitney U test was applied. Categorical distributions were compared using the chi-square test. Effect sizes are reported alongside every test, as Cliff's delta for post-level comparisons and as the standardized mean difference for paired comparisons, and *p*-values were adjusted using the Benjamini–Hochberg procedure controlling the false discovery rate at *q* = 0.05 within each of four families of comparisons: the fifteen readability and length measures, the twenty-two jargon and framing measures, the six scientificity dimensions, and the ten disciplinary shares (Benjamini and Hochberg, 1995). Because a post may be assigned to more than one discipline, the disciplinary shares were compared field by field using two-proportion z-tests rather than by a single test of independence across the ten categories. To account for possible dependence between posts written by the same account, the distribution of posts per account is reported for each platform, and the length and register comparisons were repeated on author-deduplicated corpora in which a single post per account was drawn at random, across 1,000 iterations, with median rank-biserial correlations and 2.5th to 97.5th percentile intervals reported alongside the pooled estimates. The unit of analysis and test applied to each measured quantity are summarized (Table 1).

**Table 1:** Unit of analysis, distribution and statistical test applied to each measured quantity.

Measured quantity	Unit of analysis	Distribution	Test
Field isolation	Discipline, 10 paired values	Not assessable at *n* = 10	Paired *t*-test with Wilcoxon signed-rank
Readability indices	Post	Non-normal	Mann–Whitney *U*
Linguistic density and framing	Post	Non-normal	Mann–Whitney *U*
Scientificity dimensions	Post	Non-normal	Mann–Whitney *U*
Disciplinary shares	Field counts	Categorical, multi-label	Two-proportion *z*-test per field with Benjamini–Hochberg correction
Discourse complexity classes	Post counts	Categorical	Chi-square

## 3 Results

### 3.1 Health and medicine account for the largest share of scientific discourse on both platforms

Health and medicine accounted for the largest share of field mentions on both platforms, at 23.9% on Bluesky and 19.2% on X. First we manually coded 99 posts using two human annotators was high, with a Cohen's kappa of 0.867 across all category decisions and cell-level agreement of 98.7%. Compared against the consensus labels, the automated assignment achieved a macro-averaged precision of 0.777, recall of 0.847 and F1 of 0.772, with micro-averaged values of 0.781, 0.735 and 0.758 respectively. Performance varied by category: high precision was obtained for AI and computer science (precision 1.000, recall 0.800), health and medicine (1.000, 0.571), chemistry and materials (1.000, 1.000), physics and astronomy (1.000, 1.000), and interdisciplinary and emerging topics (1.000, 0.667). Classification recall was maintained for climate and environment (0.667, 1.000), mathematics and statistics (0.500, 1.000), social sciences and psychology (0.500, 1.000), and engineering and technology (0.500, 1.000), while biology and life sciences showed lower recovery rates (0.600, 0.429). A disciplinary label was assigned to 2,704 of the 8,697 Bluesky posts and to 97,086 of the 3,48,900 X posts. Social sciences and psychology followed, at 15.9 and 14.1%, and AI and computer science accounted for 13.5 and 12.6%. Climate and environment accounted for 12.2% of field mentions on Bluesky and 8.7% on X, while mathematics and statistics accounted for 4.1 and 9.6%, engineering and technology for 4.1 and 6.8%, and physics and astronomy for 10.8 and 13.6%. Shares are expressed as proportions of all field assignments on a platform rather than of posts. Each field was compared between platforms using a two-proportion *z*-test with Benjamini–Hochberg correction across the ten fields, and seven of the ten differed: climate and environment (log2 ratio +0.49, adjusted *p* = 2.0e-11), health and medicine (+0.32, 1.4e-10) and social sciences and psychology (+0.17, 0.0058) were higher on Bluesky, while physics and astronomy (−0.33, 1.1e-05), chemistry and materials (−0.47, 0.0047), engineering and technology (−0.73, 4.4e-09) and mathematics and statistics (−1.22, *p* < 0.001) were higher on X. Biology and life sciences, AI and computer science, and interdisciplinary and emerging topics did not differ (Figure 1).

**Figure 1:**
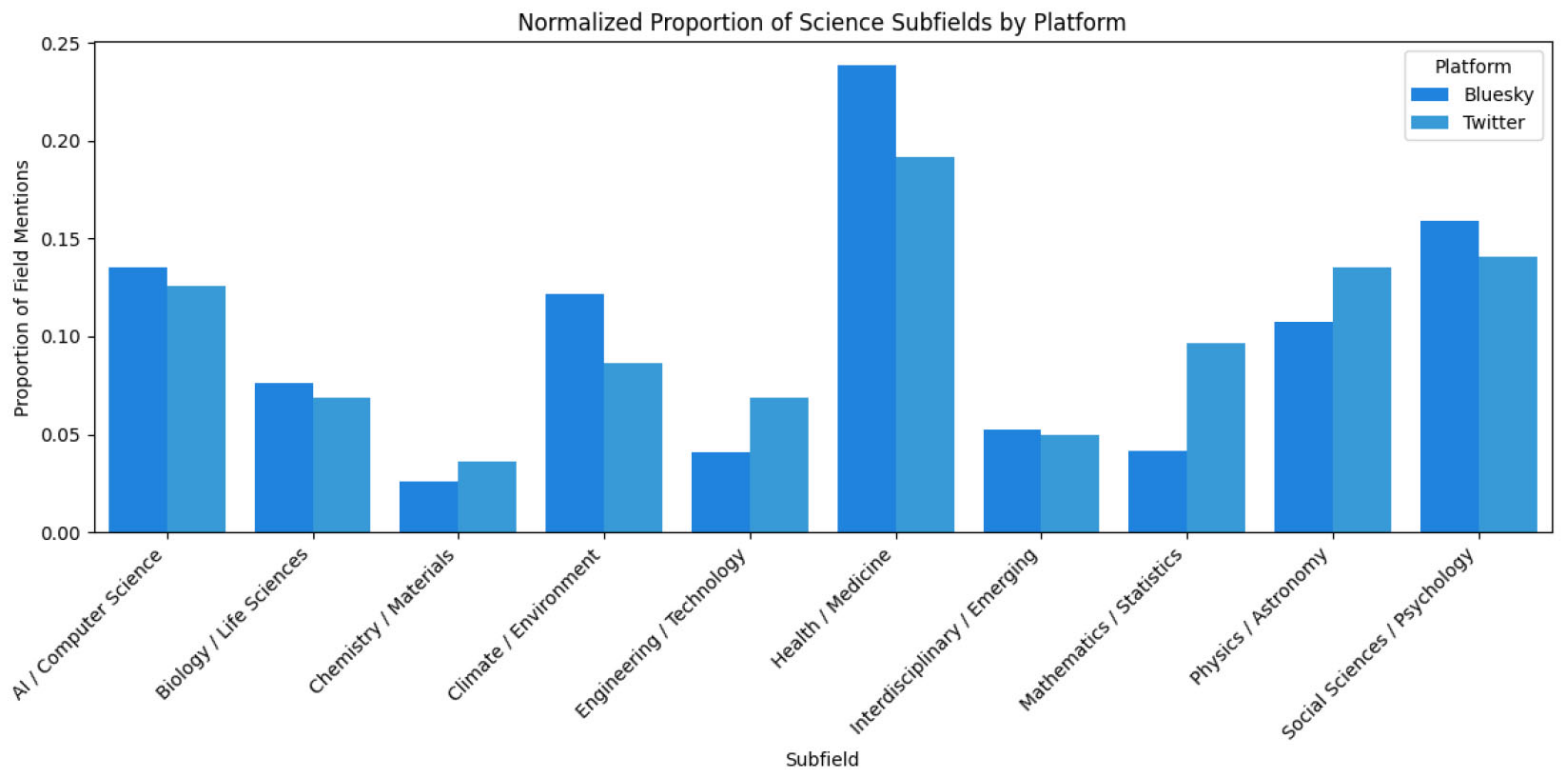
Health and medicine account for the largest share of field assignments on both platforms, with climate and environment more prominent on Bluesky and mathematics and statistics more prominent on X. Proportion of all field assignments on each platform, so that the two platforms are comparable despite differing corpus sizes. Because a post may be assigned to more than one discipline, each field was compared between platforms using a two-proportion z-test with Benjamini–Hochberg correction across the ten fields; seven of the ten differed, with climate and environment (12.2% against 8.7%, adjusted *p* = 2.0e-11), health and medicine (23.9% against 19.2%, adjusted *p* = 1.4e-10) and social sciences and psychology (15.9% against 14.1%, adjusted *p* = 0.0058) higher on Bluesky, and physics and astronomy, chemistry and materials, engineering and technology, and mathematics and statistics higher on X. *n* = 2,704 labeled Bluesky and 97,086 labeled X posts.

### 3.2 Posts referencing more than one discipline occurred more often on X than on Bluesky

Posts referencing more than one discipline accounted for 17.8% of labeled posts on X and 13.8% on Bluesky. The mean number of fields per labeled post was 1.23 on X and 1.17 on Bluesky. The strongest co-occurring pairs on Bluesky involved health and medicine with biology and life sciences, and AI and computer science with climate and environment. On X the strongest pairs involved mathematics and statistics with physics and astronomy, and mathematics and statistics with social sciences and psychology (Figure 2).

**Figure 2:**
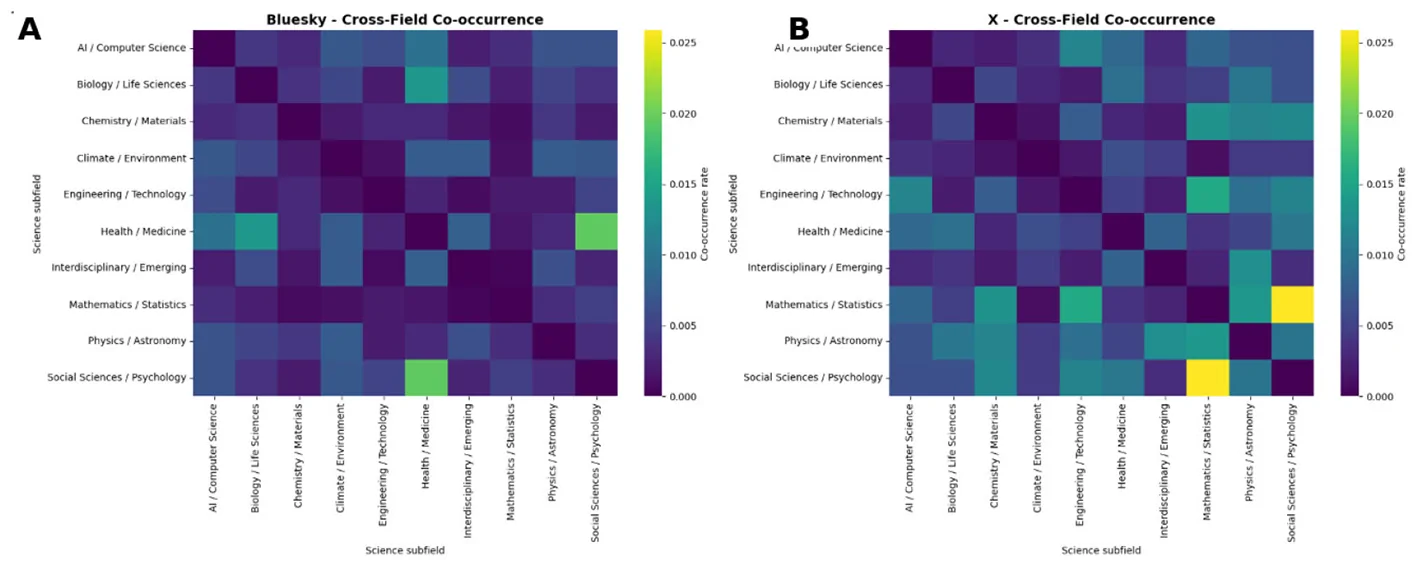
Cross-disciplinary co-occurrence is present on both platforms with different dominant pairings. Heatmaps of the rate at which pairs of scientific subfields appear together within individual posts, normalized by the number of labeled posts on each platform and plotted on a shared color scale. **(A)** Bluesky. **(B)** X.

### 3.3 Field isolation is higher on Bluesky than on X

Field isolation, the proportion of posts within a discipline that reference that discipline alone, gave an unweighted mean across the ten disciplines of 0.6882 on Bluesky and 0.6200 on X, and was higher on Bluesky in eight of the ten disciplines (paired *t*-test *t*(9) = 3.50, *p* = 0.0067; Wilcoxon signed-rank *W* = 4.0, *p* = 0.0137; standardized mean difference 1.11; Figure 3). The largest differences were in mathematics and statistics (0.695 against 0.551), engineering and technology (0.646 against 0.496) and interdisciplinary and emerging topics (0.590 against 0.479), while climate and environment and biology and life sciences showed the reverse pattern, with isolation higher on X (0.778 against 0.753 and 0.636 against 0.631). The composite disciplinary concentration index, which combines isolation, the inverse interdisciplinary rate, the inverse network density and the clustering coefficient, was 0.638 on Bluesky and 0.611 on X under equal weighting, and 0.665 and 0.633 under the weighting given in the Methods.

**Figure 3:**
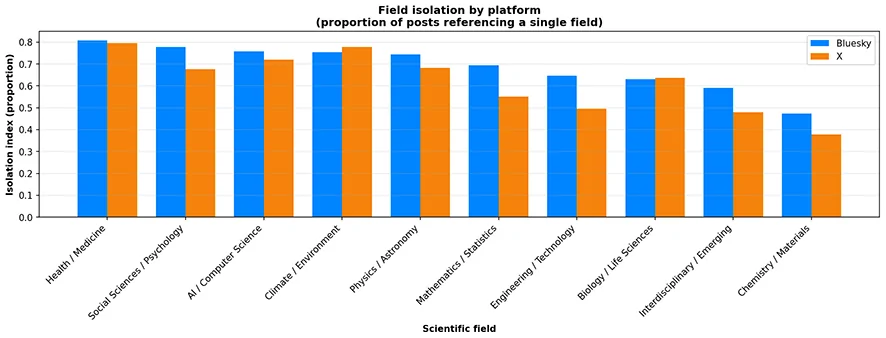
Field isolation is higher on Bluesky than on X in eight of the ten disciplines. Proportion of posts within each discipline that reference that discipline alone, calculated separately for each platform. Each discipline contributes one value per platform, giving ten paired observations, and the platforms were compared using a paired *t*-test with the Wilcoxon signed-rank test as a distribution-free confirmation (unweighted mean 0.6882 against 0.6200; *t*(9) = 3.50, *p* = 0.0067; *W* = 4.0, *p* = 0.0137; standardized mean difference 1.11). Disciplines are ordered by isolation on Bluesky. *n* = 2,704 labeled Bluesky and 97,086 labeled X posts.

### 3.4 Disciplinary diversity is marginally higher on X than on Bluesky

Shannon entropy of the field distribution was 3.045 on Bluesky and 3.172 on X, against a maximum of 3.32 for ten evenly used fields. The Simpson diversity index was 0.860 and 0.879 respectively. The interdisciplinary post rate was 13.8% on Bluesky and 17.8% on X (*z* = −5.38, *p* = 7.4e-08; Figure 4).

**Figure 4:**
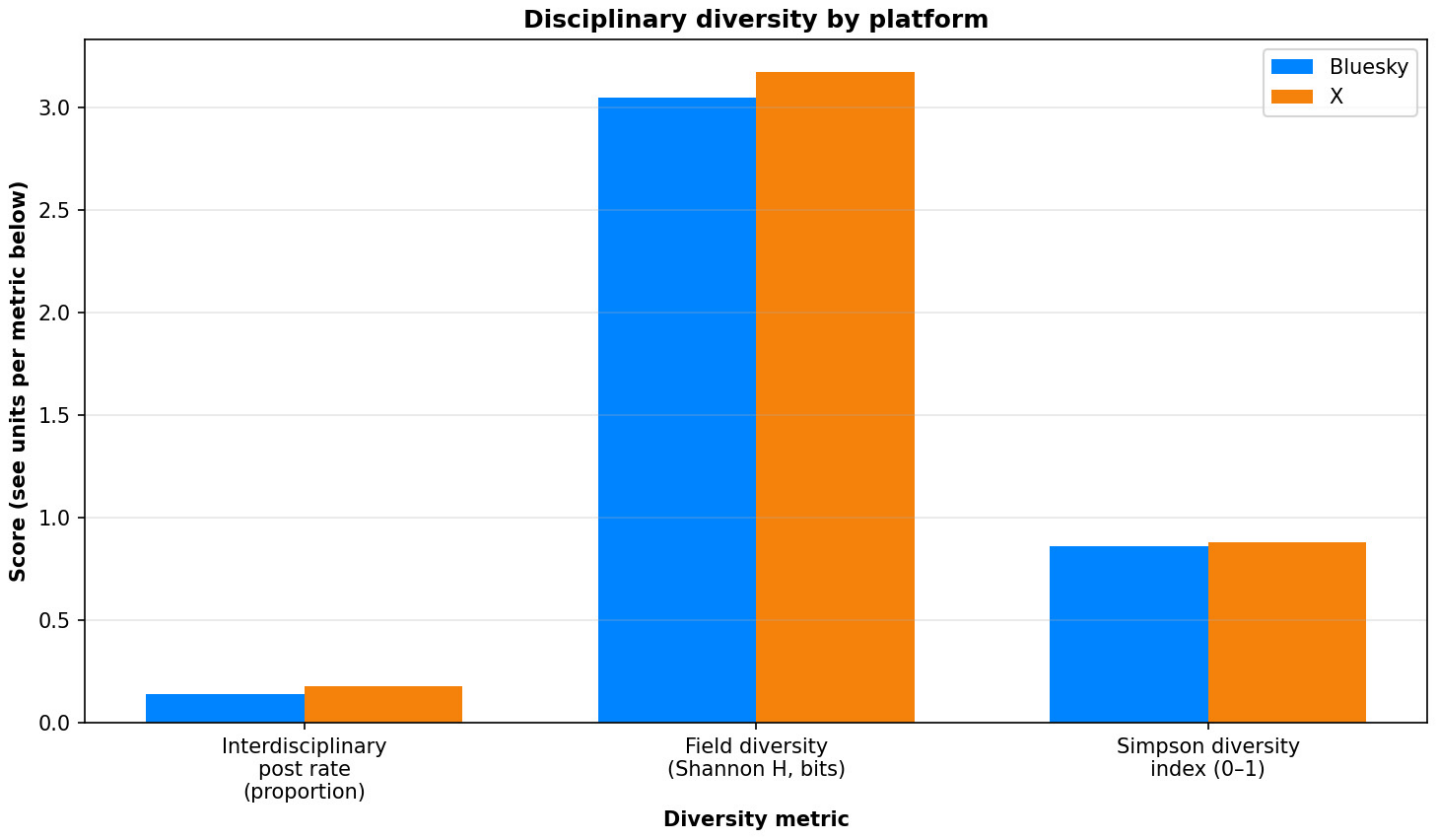
Posts on X reference more than one discipline more often than posts on Bluesky, while the two platforms show similar disciplinary diversity. Interdisciplinary post rate, Shannon entropy and the Simpson diversity index, calculated over labeled posts on each platform. The interdisciplinary post rate differed between platforms (13.8% against 17.8%, *z* = −5.38, *p* = 7.4e-08). Shannon entropy (3.045 against 3.172, against a maximum of 3.32 for ten evenly used fields) and the Simpson index (0.860 against 0.879) are single-sample summary statistics and were not tested. *n* = 2,704 labeled Bluesky and 97,086 labeled X posts.

### 3.5 The field interaction network is close to complete on both platforms

At an edge threshold of 0.05% of labeled posts, the field interaction network contained 44 of the 45 possible edges on Bluesky and all 45 on X, giving network densities of 0.978 and 1.000 and average clustering coefficients of 0.978 and 1.000. Each network formed a single connected component (Figure 5). Every pair of disciplines therefore co-occurred somewhere in both corpora, and this measure did not separate the platforms.

**Figure 5:**
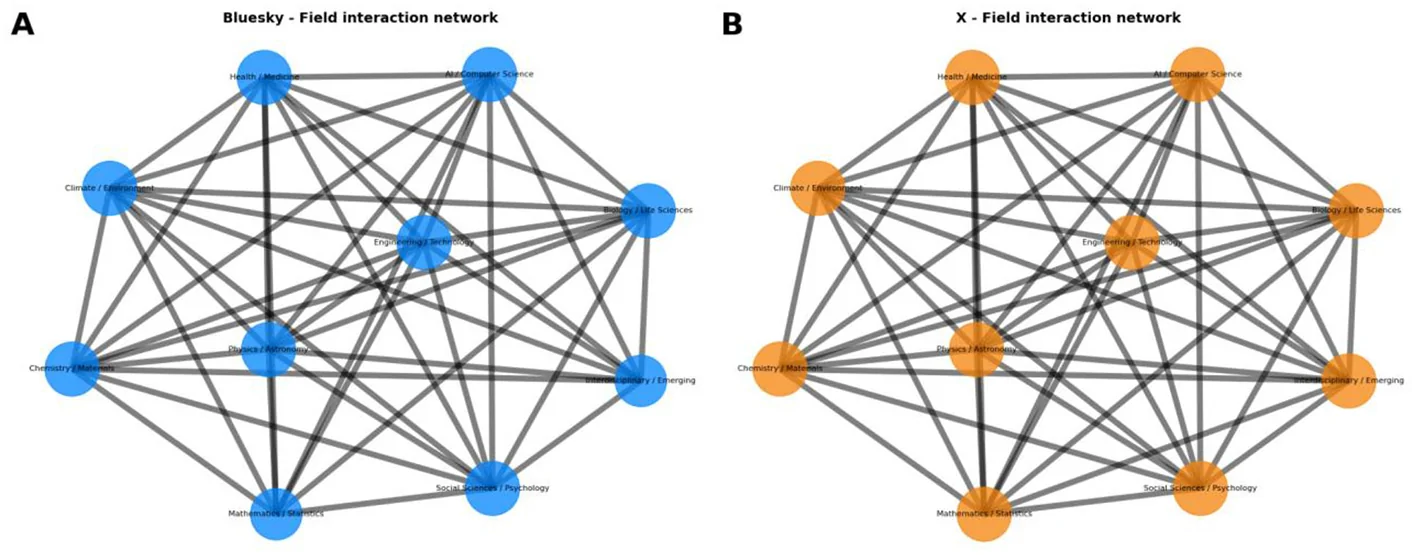
Pair of scientific subfields co-occurrence on both platforms. Field interaction networks with nodes representing the ten disciplinary categories, node size proportional to degree, edge width proportional to co-occurrence rate, and both panels drawn with the same node layout. **(A)** Bluesky. **(B)** X.

### 3.6 Bluesky posts are longer and use more complex words, while X posts use more acronyms and address readers more directly

Post-level linguistic measures differed in small but consistent ways. Bluesky posts were longer (30.6 vs. 28.7 words, adjusted *p* = 2.6e-09) and showed a higher complex word density (0.108 vs. 0.092, delta = 0.108) and a higher aggregate technical score (0.0436 vs. 0.0382, delta = 0.093), while X posts showed a higher acronym density (0.0281 vs. 0.0220) and more second-person pronouns (0.0153 vs. 0.0093, delta = −0.080) and questions per sentence (0.082 vs. 0.075; Figure 6). Bluesky posts appeared to refer to the writer more often (first-person pronoun ratio 0.0255 vs. 0.0208, delta = 0.087), whereas X posts addressed the reader more often (second-person pronouns 0.0153 vs. 0.0093, delta = −0.080). Scientific term density (0.0157 vs. 0.0152, adjusted *p* = 0.068), academic vocabulary density (0.0069 vs. 0.0075, adjusted *p* = 0.334) and noun density (0.3567 vs. 0.3523, adjusted *p* = 0.078) did not differ once correction for multiple comparisons was applied. Hedging language was more frequent on Bluesky (0.0041 vs. 0.0025, adjusted *p* = 4.8e-06). Among the remaining measures of subjectivity and emotional language, sentiment polarity (0.0963 vs. 0.0791, adjusted *p* = 8.2e-05) and exclamations per sentence (0.1057 vs. 0.0774, adjusted *p* = 2.0e-07) were higher on Bluesky, while emotional verbs (0.0052 vs. 0.0054), storytelling language (0.0035 vs. 0.0038), certainty language (0.0007 vs. 0.0008), passive constructions per sentence (0.1677 vs. 0.1702) and sentiment subjectivity (0.4001 vs. 0.3940) did not differ after correction. All effect sizes were small, with the largest Cliff's delta across the comparison being 0.142.

**Figure 6:**
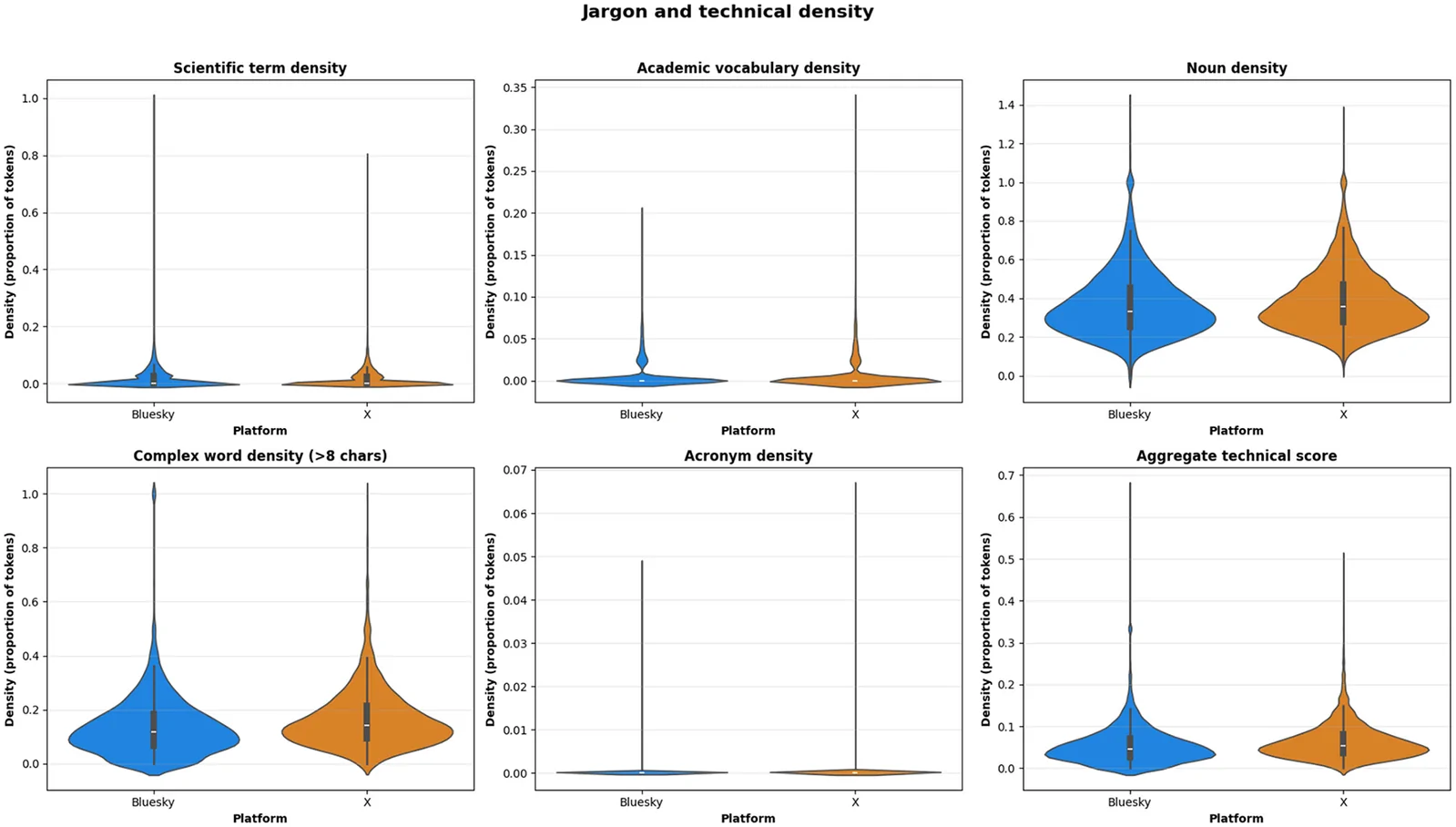
Complex word density and the aggregate technical score are higher on Bluesky and acronym density is higher on X, while scientific term density, academic vocabulary density and noun density do not differ between platforms. Distributions of jargon and technical density measures per post, expressed as a proportion of tokens. **(A)** Scientific term density (0.0157 against 0.0152, adjusted *p* = 0.068). **(B)** Academic vocabulary density (0.0069 against 0.0075, adjusted *p* = 0.334). **(C)** Noun density (0.3567 against 0.3523, adjusted *p* = 0.078). **(D)** Complex word density (0.1082 against 0.0921, adjusted *p* = 1.8e-23). **(E)** Acronym density (0.0220 against 0.0281, adjusted *p* = 2.8e-05). **(F)** Aggregate technical score (0.0436 against 0.0382, adjusted *p* = 3.7e-19). Values are Bluesky against X. Comparisons used the Mann–Whitney *U* test with Benjamini–Hochberg correction across the family of twenty-two jargon and framing measures. *n* = 4,867 Bluesky and 4,809 X posts.

### 3.7 Readability indices place Bluesky slightly above X, and Bluesky posts fall more often within the academic band

Readability measures suggested a small difference in accessibility. The fifteen measures examined pointed in a consistent direction after correction for multiple comparisons, with the grade-level indices placing Bluesky slightly higher: Flesch-Kincaid 10.02 against 9.07, Gunning Fog 12.12 against 10.98, SMOG 11.35 against 10.49, Coleman-Liau 11.28 against 10.29 and the Automated Readability Index 11.04 against 9.59, with Flesch Reading Ease correspondingly lower on Bluesky at 51.14 against 55.37 (Table 2). The underlying components appeared to follow the same pattern, with longer words (5.23 against 5.08 characters), more syllables per word (1.62 against 1.58), longer sentences (15.98 against 14.54 words) and a higher polysyllabic word ratio (0.156 against 0.142). The proportion of posts falling within the academic band was 16.6% on Bluesky and 12.7% on X, while the proportion within the accessible band was 7.3 and 10.7%, with the great majority of posts on both platforms falling within the intermediate band (76.1 and 76.6%; Figure 7). Effect sizes were small throughout, with Cliff's delta ranging from 0.044 to 0.156, so these differences are best read as tendencies rather than as categorical distinctions. The distribution across the three complexity classes differed between platforms (chi-square (2) = 100.53, *p* = 1.5e-22).

**Table 2:** Readability metrics comparison between Bluesky and X.

Metric	Bluesky mean	X mean	Cliff's delta	Adjusted *p*
Flesch reading ease (higher = easier)	51.14	55.37	−0.103	2.9e-31
Flesch-Kincaid grade	10.02	9.07	+0.121	1.5e-39
Gunning fog index	12.12	10.98	+0.125	9.4e-44
SMOG index	11.35	10.49	+0.127	7.7e-45
Coleman-Liau index	11.28	10.29	+0.121	7.7e-44
Automated readability index	11.04	9.59	+0.156	6.5e-67
Dale-Chall score	10.97	10.83	+0.044	5.6e-06
Difficult words (count)	8.64	7.80	+0.105	3.6e-34
Difficult words (proportion)	0.305	0.296	+0.056	1.2e-11
Characters per word	5.23	5.08	+0.103	4.4e-36
Syllables per word	1.62	1.58	+0.087	2.0e-25
Words per sentence	15.98	14.54	+0.109	2.3e-28
Type-token ratio	0.914	0.921	−0.046	1.5e-10
Polysyllabic word ratio	0.156	0.142	+0.088	9.8e-26

**Figure 7:**
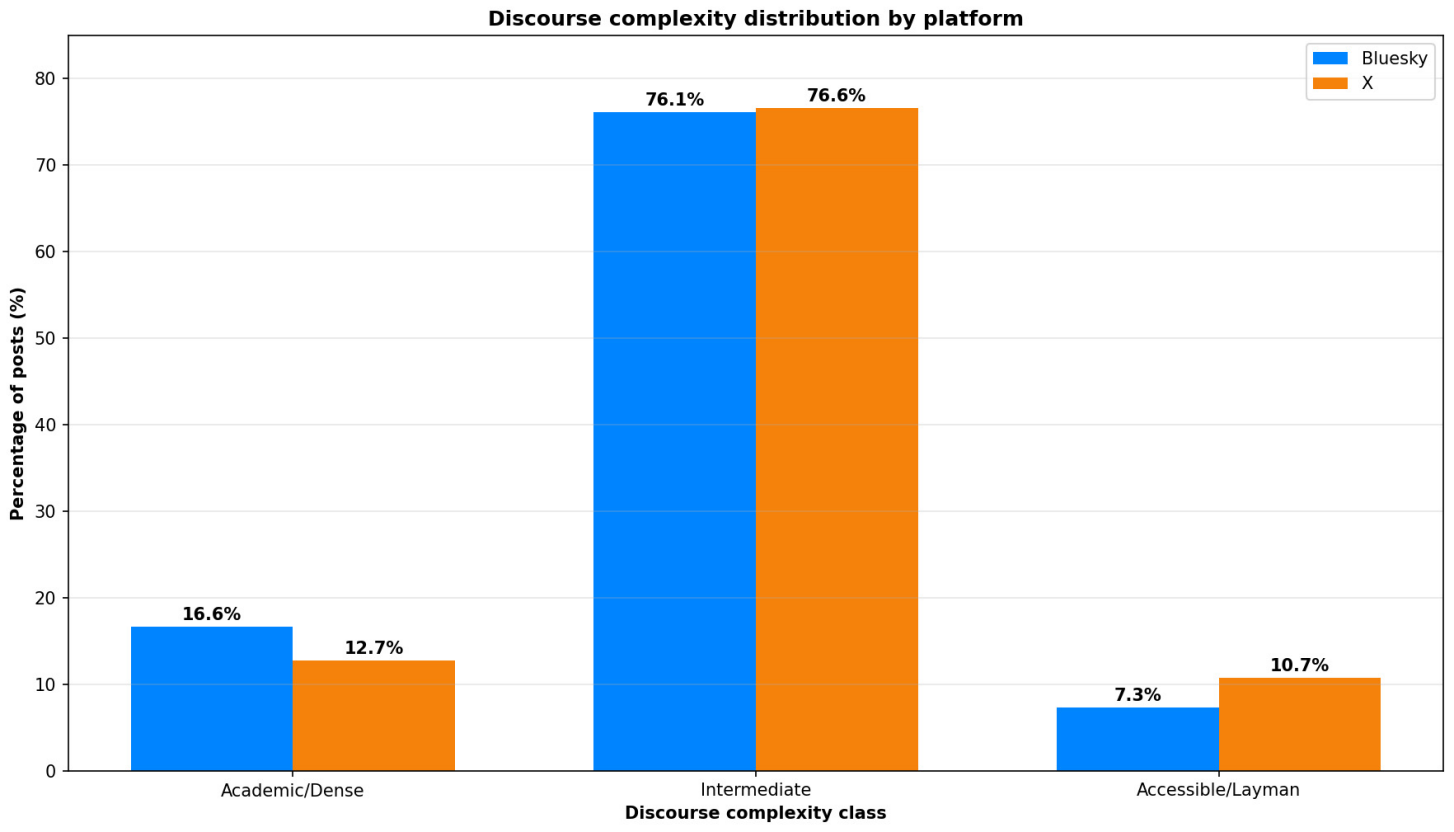
Relative to X, Bluesky shows a larger proportion of posts in the academic complexity class and a smaller proportion in the accessible class, while the proportion in the intermediate class is similar on both platforms. The three classes were distributed differently between platforms (chi-square (2) = 100.53, *p* = 1.5e-22), with differences in the academic class (16.6% against 12.7%, *z* = 7.15, *p* = 8.7e-13) and the accessible class (7.3% against 10.7%, *z* = −7.69, *p* = 1.5e-14) but not the intermediate class (76.1% against 76.6%, *z* = −0.76, *p* = 0.449). *n* = 8,448 Bluesky and 8,358 X posts.

### 3.8 Composite scientificity is similar between platforms and the six dimensions do not form a single construct

The Composite Scientificity Index, which aggregates six markers of scholarly phrasing and is distinct from the readability measures reported above, showed little separation between platforms (0.0098 on Bluesky and −0.0100 on X, Mann–Whitney U *p* = 0.264, rank-biserial 0.013). Bluesky was higher on four of the six contributing dimensions, of which only epistemic hedging differed after correction (0.0039 vs. 0.0024, adjusted *p* = 5.2e-04), while argument structure and emotion suppression were marginally higher on X (Figure 8). Removing each dimension in turn preserved the direction of the difference in every case, with the magnitude changing between −88% when epistemic hedging was removed and +53% when emotion suppression was removed. Inter-dimension correlations did not exceed 0.10, and principal component analysis gave components explaining between 19.2 and 14.7% of variance, with mixed loadings on the first component. The six dimensions therefore appear to vary largely independently, and the index is best read as a summary of distinct measures rather than as a unidimensional scale.

**Figure 8:**
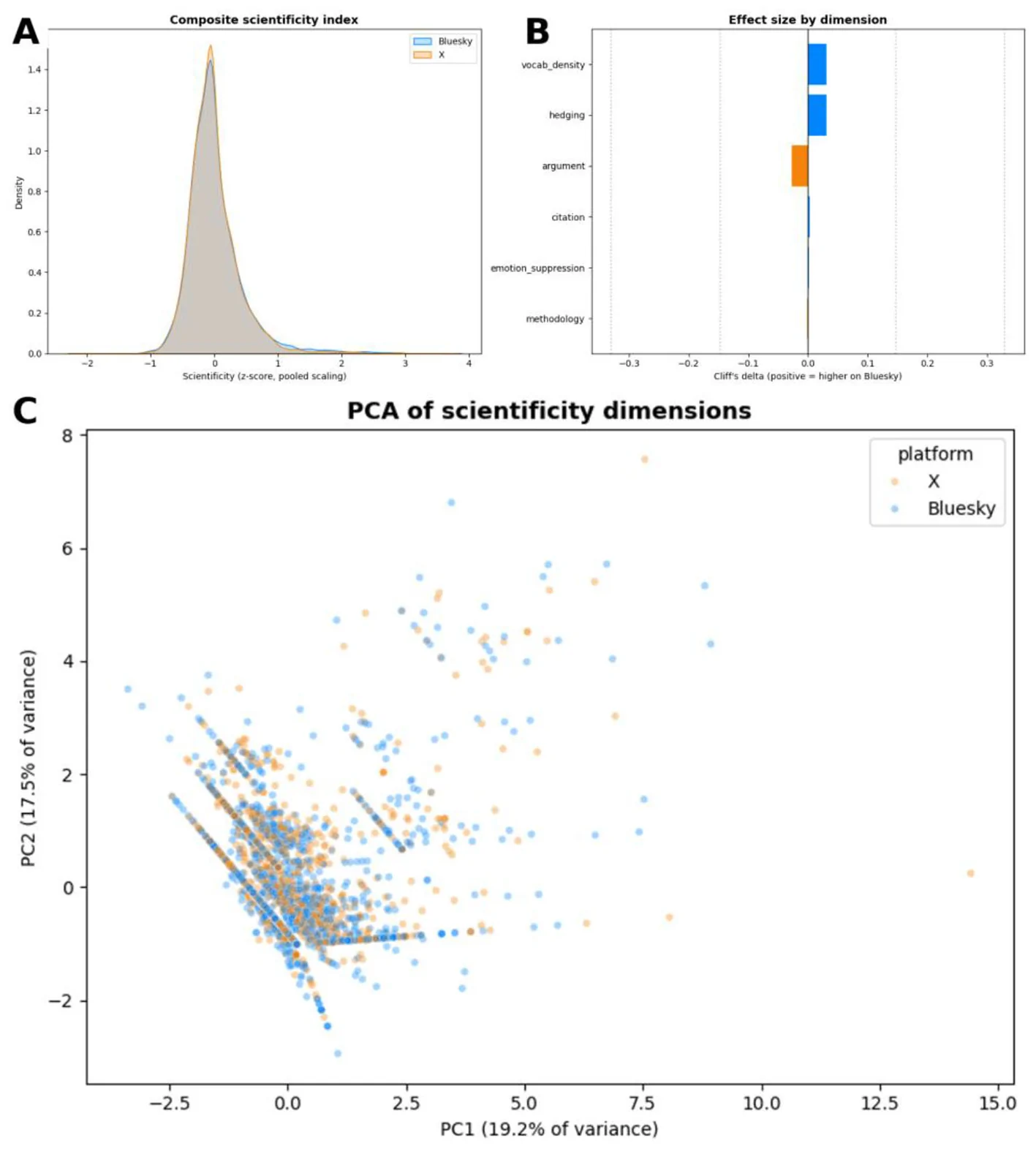
The scientificity dimensions do not form a single underlying construct. **(A)** Distribution of the Composite Scientificity Index on each platform, expressed as a z-score against the pooled sample. **(B)** Cliff's delta for each of the six dimensions, positive values indicating higher values on Bluesky. **(C)** Posts from both platforms projected onto the first two principal components of the six dimensions, which explain 19.2 and 17.5% of the variance respectively.

### 3.9 Nine of eleven metrics investigated remain outside the range of size-matched X subsamples

One hundred random subsamples of the X corpus, each matched in size to the Bluesky corpus, were drawn without replacement and eleven metrics were recomputed on each. The Bluesky value fell outside the 2.5th to 97.5th percentile range of the X subsamples for nine of the eleven metrics: field isolation (0.6882 against a subsample range of 0.5997 to 0.6447), interdisciplinary rate (0.1376 against 0.1633 to 0.1914), Shannon entropy (3.0451 against 3.1511 to 3.1881), Simpson index (0.8603 against 0.8755 to 0.8815), complex word density (0.1065 against 0.0897 to 0.0928, computed over the full Bluesky corpus), acronym density (0.0216 against 0.0246 to 0.0276), hedging language (0.0039 against 0.0025 to 0.0029), aggregate technical score (0.0428 against 0.0371 to 0.0384) and Flesch-Kincaid grade (10.01 against 8.95 to 9.12). Scientific term density and academic vocabulary density fell within the subsample range, in agreement with the post-level comparisons in which neither differed after correction for multiple comparisons. Values in this analysis are computed over the full Bluesky corpus rather than the matched samples of 5,000 posts used for the post-level comparisons, which accounts for small differences from the corresponding figures reported above. Full per-metric results are given in Supplementary Material.

### 3.10 Length and register differences between the platforms hold when each account contributes a single post

To investigate whether prolific accounts influenced the difference in length and register between the two platforms, random samples of 5,000 posts were drawn from each of the 8,697 Bluesky and 3,48,900 X posts. Posts containing fewer than five tokens were excluded from feature extraction, as the per-token and per-sentence ratios are unstable at that length, leaving 4,867 Bluesky posts written by 4,070 accounts and 4,809 X posts written by 4,553 accounts. The most prolific 1% of accounts contributed 8.5% of posts on Bluesky and 4.4% on X, and the largest single account contributed 61 and 57 posts respectively. Ten length and register measures were then recompared on author-deduplicated corpora, in which one post per account was drawn at random, across 1,000 iterations. The 2.5th to 97.5th percentile interval of the rank-biserial correlation excluded zero for all ten measures, and the median correlations and their intervals are given for each measure (Table 3). Positive values indicate that Bluesky ranked higher.

**Table 3:** Author-deduplicated comparison of length and register measures, 1000 iterations.

Measure	Median *r*	2.5th	97.5th
Words per sentence	+0.139	+0.136	+0.143
Characters per word	+0.078	+0.075	+0.081
Words per post	+0.097	+0.094	+0.100
Hashtags per post	+0.047	+0.045	+0.048
Negation ratio	−0.061	−0.064	−0.058
Questions per sentence	−0.045	−0.047	−0.042
Exclamations per sentence	+0.053	+0.052	+0.055
Lexical polarity	+0.033	+0.030	+0.035
Type-token ratio	−0.039	−0.043	−0.035
Positive affect	+0.033	+0.031	+0.035

## 4 Discussion

Scientific discourse on Bluesky and on X seems to share various disciplines, albeit with different frequencies. Health and medicine led on both platforms, at 23.9 and 19.2% of field mentions, followed on both by social sciences and psychology and by AI and computer science, a profile also reported for Bluesky in an independent analysis of 2.6 million posts referencing scholarly articles (Zhang M. et al., 2025). Climate and environment was higher on Bluesky, at 12.2% against 8.7%, while mathematics and statistics was higher on X, at 9.6% against 4.1%, as were engineering and technology and physics and astronomy (Figure 1). Seven of the ten fields investigated differed in share after correction for multiple comparisons, with the largest differences in climate and environment and in mathematics and statistics, while the three remaining fields did not differ. However, overall, the two platforms seems to share the same scientific disciplines.

Posts on X referenced more than one discipline more frequently and addressed the reader more directly than posts on Bluesky. Posts referencing more than one field accounted for 17.8% of labeled posts on X against 13.8% on Bluesky (*z* = −5.38, *p* = 7.4e-08; Figure 4). Second-person pronouns were used at 0.0153 against 0.0093 and questions were more frequent per sentence (Figure 6), and 10.7% of X posts fell in the accessible band against 7.3% on Bluesky (Figure 7). Simplified vocabulary and direct address to the reader have been described as characteristics of science communication intended for non-specialist audiences (Bubela et al., 2009). Cote and Darling reported that researchers with larger followings on X reached audiences containing substantial proportions of non-scientists, whereas those with smaller followings were read mainly by other researchers (Cote and Darling, 2018). These observations are consistent with a mode of communication on X in which posts are formulated for readers who are not assumed to be specialists in the field under discussion.

Posts on Bluesky referenced a single discipline more frequently and were written at a higher level of linguistic complexity than posts on X. Field isolation, the proportion of posts within a discipline referencing that discipline alone, gave an unweighted mean across the ten disciplines of 0.6882 on Bluesky against 0.6200 on X and was higher on Bluesky in eight of the ten disciplines (paired *t*-test t(9) = 3.50, *p* = 0.0067; Wilcoxon signed-rank *W* = 4.0, *p* = 0.0137; Figure 3). Disciplinary concentration measured in the content of posts is distinct from restricted exposure to opposing views, which is the defining feature of an echo chamber (Cinelli et al., 2021; Nguyen, 2020). Sentences averaged 15.98 words against 14.54, words averaged 5.23 characters against 5.08, and 16.6% of posts fell in the academic band against 12.7% (Table 2, Figure 7). First-person pronouns were used at 0.0255 against 0.0208 (Figure 6). Self-mention has been described as a means by which authors claim credit for their contribution and establish a position within a disciplinary community, and its frequency has been reported to vary with the extent to which a text is addressed to specialist readers (Hyland, 2001, 2002). Scholarly posts on Bluesky have also been reported to show higher textual originality and a lower proportion of reposts than equivalent posts on X (Zheng E-T. et al., 2025; Fieldhouse, 2025). These observations are consistent with a peer-oriented register on Bluesky, in which posts are formulated for readers already working within the field under discussion, although interaction between accounts was not measured.

The greater linguistic complexity of Bluesky posts was not accompanied by a higher density of scholarly markers. The Composite Scientificity Index did not separate the platforms (0.0098 against −0.0100, *p* = 0.264), and of its six dimensions only epistemic hedging differed after correction. Scientific term density, academic vocabulary density and noun density did not differ once correction for multiple comparisons was applied, so the complexity difference rests on word and sentence length rather than on technical vocabulary. No pair of dimensions correlated above 0.10 and no principal component explained more than 19.2% of the variance, which indicates that vocabulary, methodology, citation, epistemic hedging, argumentation and emotional restraint vary independently in short social media posts. Composite measures of scientific communication should therefore be reported alongside their components, since averaging dimensions that do not covary summarizes rather than measures a single underlying property (Jeong et al., 2024). The difference between the platforms therefore lies in how much is written and how it is phrased, not in how much specialist terminology is used.

Taken together, these findings indicate that although Bluesky and X cover the same disciplines at differing frequencies, the two platforms differ in the way scientific posts are formulated. Posts on X were shorter, used more accessible language and addressed the reader directly, whereas posts on Bluesky were longer, used longer words and sentences and were more often confined to a single discipline, with a higher frequency of self-mention. The difference in complexity seems to arise from word and sentence length rather than from the density of technical vocabulary, which did not differ significantly between the platforms. Audience responses to scientific content have been reported to depend on the identity of the source as well as on the content itself and emotionally amplified material has been observed to spread readily on X (Zhang and Lu, 2023; Wu et al., 2025). Migration of researchers from X to Bluesky has been documented in survey and poll data, with a majority of respondents to a journal poll reporting use of the platform and researchers describing X as no longer professionally useful (Biever, 2025; Shiffman and Wester, 2025). We hypothesize that the two registers continue to diverge, scientific discussion conducted in the more demanding register may become less accessible to non-specialist readers and can affect dissipation of informed scinetfic content to the wider community (Bubela et al., 2009).

## 5 Limitations

Retrieval was based on the single term science, which serves as an approximation of scientific discourse rather than an exhaustive sampling frame. Field assignment further depends on a post containing one of the listed keywords, so the labeled subset comprises posts that name their discipline explicitly and does not include posts discussing a field in vocabulary absent from the dictionary. Posts discussing specialized research without using the generic term may therefore not be captured, which may bias both corpora toward posts addressing science reflexively and away from technical exchanges between specialists, although the comparison remains internally consistent because the same term was applied to both platforms. The disciplinary and linguistic profile of Bluesky may also reflect the current developmental stage of the platform rather than a stable property of it, since its scientific user base was assembled largely through targeted migration and starter packs, which favor dense and self-selected professional communities (Balduf et al., 2025; Jeong et al., 2024). As the user base broadens, the engagement dynamics documented on mature platforms may attenuate the differences observed here (Wu et al., 2025), and the present comparison is therefore best read as a snapshot of two platforms at different points of maturity. It is also worth mentioning that, the automated classification identifies disciplines using predefined keywords rather than context. It therefore captures only posts containing disciplinary terms from the dictionary and may assign labels that a human coder would not. The findings should thus be interpreted as describing this classified subset rather than all science-related posts. However, cross-platform comparisons remain consistent because the same procedure was applied to both corpora.

## 6 Conclusion

Overall, posts on Bluesky appeared more complex, more academic, and higher in first-person pronoun use, whereas posts on X were more audience-oriented, more diverse in the disciplines a single post referenced, and easier to read. Bluesky's posting patterns suggest that its content is confined to discrete messages designed to resonate with peers within the scientific community, whereas X posts seem constrained by the goal of driving high engagement.

## Data Availability

The original contributions presented in the study are included in the article/Supplementary material, further inquiries can be directed to the corresponding authors.
