# Supplementary material for: Differences in disciplinary concentration and linguistic complexity between scientific posts on Bluesky and X
Source: Front Res Metr Anal. 2026 Sep 28;11:1912970. doi: 10.3389/frma.2026.1912970 (PMC13655012; doi:10.3389/frma.2026.1912970)
Supplement: Supplementary file 1 [file Data_Sheet_1.docx]

**Supplementary Material**

Differences in Disciplinary Concentration and Linguistic Complexity Between Scientific Posts on Bluesky and X

**Supplementary File 1. Per-metric results of the sensitivity analysis**

One hundred random subsamples of the X corpus, each matched in size to the Bluesky corpus of 8,697 posts, were drawn without replacement and eleven metrics recomputed on each. The Bluesky point estimate is compared against the resulting distribution of X subsample values. The final column indicates whether the Bluesky value falls outside the 2.5th to 97.5th percentile range of the X subsamples.

| **Metric** | **Bluesky** | **X subsample mean** | **X 2.5th** | **X 97.5th** | **Outside interval** |
| --- | --- | --- | --- | --- | --- |
| Field isolation index | 0.6882 | 0.6207 | 0.5997 | 0.6447 | Yes |
| Interdisciplinary post rate | 0.1376 | 0.1770 | 0.1633 | 0.1914 | Yes |
| Shannon entropy (H) | 3.0451 | 3.1686 | 3.1511 | 3.1881 | Yes |
| Simpson diversity index | 0.8603 | 0.8787 | 0.8755 | 0.8815 | Yes |
| Scientific term density | 0.0151 | 0.0150 | 0.0144 | 0.0156 | No |
| Academic vocabulary density | 0.0069 | 0.0069 | 0.0065 | 0.0073 | No |
| Complex word density | 0.1065 | 0.0912 | 0.0897 | 0.0928 | Yes |
| Acronym density | 0.0216 | 0.0262 | 0.0246 | 0.0276 | Yes |
| Hedging language | 0.0039 | 0.0027 | 0.0025 | 0.0029 | Yes |
| Aggregate technical score | 0.0428 | 0.0377 | 0.0371 | 0.0384 | Yes |
| Flesch-Kincaid grade | 10.0111 | 9.0411 | 8.9473 | 9.1194 | Yes |

**Supplementary File 2. Disciplinary keyword dictionaries**

Keyword dictionaries used for the rule-based disciplinary assignment. Each post was scanned for matches at word boundaries, so that a keyword is counted only where it occurs as a complete word rather than as a substring of a longer word, and posts were allowed to match more than one category.

| **Category** | **n terms** | **Keywords** |
| --- | --- | --- |
| AI / Computer Science | 10 | AI; artificial intelligence; machine learning; deep learning; neural network; algorithm; data science; computer vision; natural language processing; NLP |
| Health / Medicine | 16 | covid vaccine; vaccine; health; medicine; disease; immunology; genetics; cancer; cardiology; neuroscience; mental health; epidemiology; therapy; clinical trial; pharmacology; biomedicine |
| Biology / Life Sciences | 16 | biology; microbiology; cell; genome; genomics; proteomics; gene editing; crispr; ecology; evolution; biodiversity; neurobiology; plant science; zoology; molecular biology; microbiome |
| Chemistry / Materials | 10 | chemistry; chemical; organic chemistry; inorganic chemistry; polymer; nanomaterials; catalyst; biochemistry; materials science; nanotech |
| Physics / Astronomy | 15 | physics; quantum; particle; relativity; cosmology; astrophysics; black hole; dark matter; string theory; optics; mechanics; thermodynamics; space; satellite; astronomy |
| Climate / Environment | 14 | climate change; climate; global warming; carbon; environment; sustainability; pollution; earthquake; geology; oceanography; meteorology; renewable energy; climate policy; green energy |
| Mathematics / Statistics | 10 | math; mathematics; statistics; probability; algebra; calculus; geometry; number theory; topology; data analysis |
| Social Sciences / Psychology | 9 | psychology; sociology; economics; behavior; cognition; education; political science; anthropology; neuropsychology |
| Engineering / Technology | 11 | engineering; electrical; mechanical; civil; software; hardware; automation; robotics; bioengineering; virtual reality; augmented reality |
| Interdisciplinary / Emerging | 16 | synthetic biology; quantum computing; bioinformatics; systems biology; computational biology; environmental science; photonics; ai ethics; data privacy; space science; virus; bacteria; nutrition; geography; ocean; biotech |

**Supplementary File 3. Most frequent nouns in biology and life sciences posts**

Word clouds of the most frequent nouns in posts assigned to biology and life sciences on each platform, generated with a maximum of 50 words displayed. Part-of-speech tagging was performed with spaCy, and URLs, mentions and hashtags were removed before tagging. (A) Bluesky. (B) X.
